# Synthesis and electrochemical properties of Mn-doped Li_2_Mn_0.1_Ti_1.9_(PO_4_)_3_ materials

**DOI:** 10.3389/fchem.2023.1189866

**Published:** 2023-06-01

**Authors:** Lixia Sun, Jiayan Hu, Weiqi Bai, Wutao Mao, Zhongcheng Song

**Affiliations:** School of Chemistry and Chemical Engineering, Jiangsu University of Technology, Changzhou, China

**Keywords:** Mn-doped, Li_2_Mn_0.1_Ti_1.9_(PO_4_)_3_, LiTi_2_(PO_4_)_3_, Li_2_Ti_2_(PO_4_)_3_, electrochemical property

## Abstract

The hunt for a higher power storage, relatively inexpensive, non-polluting battery technology is currently a pressing issue because of the rapid growth of the worldwide economic and the progressively significant environmental pollution. Among the possible nanomaterials for rechargeable batteries that can have heteroatoms applied to it in order to improve its electrochemical behavior is Li_x_Ti_y_(PO_4_)_3_. Carbon-coated Mn-doped Li_2_Mn_0.1_Ti_1.9_(PO_4_)_3_ materials was synthesized by spray drying method. The material was characterized by XRD, SEM, TEM, BET, TGA et al. Crystal data refinement results by Rietveld method showed that the symmetry space group is Pbcn.The lattice parameters of Li_2_Mn_0.1_Ti_1.9_(PO_4_)_3_ are *a* = 11.9372 Å, *b* = 8.5409 Å, *c* = 8.5979 Å, *α* = *β* = *γ* = 90°, *V* = 876.59 Å^3^ and *Z* = 4). Rietveld refinement was performed, and the confidence factors are Rwp = 11.79%, Rp = 9.14%, and χ^2^ = 1.425. It was exhibited that LMTP0.1/CA-700 material has good crystallinity. Testing the cells with LAND test procedure (200 mA/g current density for 200 cycles), the LMTP0.1/CA-700 material has a discharge specific capacity of about 65 mAh/g. The capacity decayed by only 3% during the cycle. It has some potential application values as cathode of lithium ion battery in the future.

## 1 Introduction

At present, with the rapid development of the global economy and the increasingly serious environmental pollution, the search for a high energy storage, low-cost, non-polluting battery device has become an urgent matter (Gao & Yang, 2010; Jin et al., 2022; Sun et al., 2022). Various ways to improve the energy storage of the material have been reported in the literature. LiTi_2_(PO_4_)_3_ material is one of lithium-ion battery materials that can be added by adding heteroatoms to the material with a view to improving the electrochemical properties of the material (He et al., 2016; Giarola et al., 2017; Weng et al., 2017; Kwatek et al., 2018; Pang et al., 2018; Zhou et al., 2018; Guo et al., 2022). The doped elements can enhance the cycling performance of the material by enhancing the transport between electrons or ions (Zhao et al., 1994; Capsoni et al., 2010; Sun et al., 2015; Wang et al., 2017; He et al., 2018; Jiang et al., 2019a; He & Xu, 2019; Wang et al, 2020; Luo et al., 2020; Luo et al., 2023; Sradhasagar et al., 2023). Liang *et al.* prepared Ga-doped LiTi_2_(PO_4_)_3_ materials by hydrothermal method and showed that the electrochemical performance of the material was greatly improved after doping with Ga elements, which may be due to the formation of a dense layer after doping with elements, which requires less activation energy and makes the reaction easier (Liang et al., 2019). Jiang *et al.* prepared K-doped LiTi_2_(PO_4_)_3_ using sol-gel method materials, and the results showed that the doped elements did not change the original lattice structure (Jiang et al., 2019b). The material had the best performance when the doping ratio of K was 0.03, probably because the appropriate doping amount facilitated the expansion of Li^+^ mobility. Zhang *et al.* synthesized Al and Fe co-doped LiTi_2_(PO_4_)_3_ materials by the sol-gel method, and the results showed that the material was more stable in saturated aqueous LiOH solution after co-doping (Zhang et al., 2014).

Mn^2+^ doping has shown high electrochemical performance applications in some battery studies, such as Mn-doped FeS (Chen et al., 2022), Mn-doped LiFePO_4_(Gupta et al., 2017), Mn-doped LiCoPO_4_(Lin et al., 2011), Mn-doped K_0.23_V_2_O_5_(Shi et al., 2021), Mn-doped LiPON electrolyte (Song et al., 2023), Mn-doped V_2_O_5_(Zeng et al., 2015), Mn-doped Co_3_O_4_(Zhang et al., 2023) et al. Li_2_Ti_2_(PO_4_)_3_ (Kee et al., 2011) has the same crystal structure as combined-metallic compounds as Li_2_TiM(PO_4_)_3_ (M = Fe, Cr) (Patoux et al., 2004) and Li_2_FeZr(PO_4_)_3_ (Catti, 2001), which have been extensively studied as potential secondary battery materials (Yin et al., 2003). In this paper, carbon coated Mn-doped Li_2_Mn_0.1_Ti_1.9_(PO_4_)_3_ materials were successfully prepared by spray drying method using anhydrous citric acid as the carbon source and the effect of sintering temperature on the material properties was investigated. The synthesized materials were subjected to a series of characterizations such as XRD, SEM and TEM to test the morphological structure of the materials. Cells with different electrolyte systems were assembled and tested for their electrochemical properties.

## 2 Material and methods

### 2.1 Materials and synthesis

Lithium dihydrogen phosphate (LiH_2_PO_4_), lithium acetate (CH_3_COOLi⋅H_2_O), manganese acetate (Mn(CH_3_COO)_2_⋅2H_2_O) and Dihydroxybis (ammonium lactato)titanium IV) (Ti(OH)_2_(OCH(CH_3_)COONH_4_)_2_) aqueous solution as raw materials. All starting materials were purchased from Sinopharm Chemical Reagent Co., Ltd (Shanghai, China) and Shanghai Aladdin Biochemical Technology Co., Ltd (Shanghai, China). LiH_2_PO_4_, CH_3_COOLi⋅H_2_O and Mn(CH_3_COO)_2_⋅2H_2_O were dissolved in 300 mL of pure water according to a certain stoichiometric ratio, and the corresponding proportion of aqueous Dihydroxybis (ammonium lactato)titanium IV) solution was added under stirring conditions. After stirring for 30 min, spray drying was carried out. The spray drying program was set to 240°C at the inlet and 15 rmp at the feed rate, and the feed was started after the outlet temperature was above 85°C. After spray drying, the precursor material is obtained, and the precursor material and carbon source anhydrous citric acid are weighed and mixed in a certain ratio. The mixed material was added to the porcelain boat and heated up to 600°C, 700°C and 800°C in a tube furnace at a certain heating rate (4 °C/min) for 4 h. The final black solid powder (noted as LMTP0.1/CA-600, LMTP0.1/CA-700, LMTP0.1/CA-800, respectively) was obtained.

### 2.2 Material characterization

An X-ray diffractometer with model X'PERT POWDER was used to analyze the crystallinity and composition of the material. Before sample preparation, the sample needs to be ground and then laid flat on the groove of the substrate for easy testing. Test conditions: cathode with Cu target Ka radiation, scanning range of 10°–80°, scanning speed of 8°/min. S-3400N scanning electron microscope (SEM, from Hitachi) was used to observe the morphology of the material. High-resolution transmission electron microscopy (HRTEM, from JEM-2100) were used to research the microstructure of the samples. The carbon content of the sample can be known by Thermogravimetric Analysis (TGA). X-ray photoelectron spectroscopy (XPS, from Nexsa) to indicate the structural valence of the materials.

### 2.3 Electrochemical properties testing

#### 2.3.1 Preparation of carbon-coated LMTP negative electrode material

The carbon-coated LMTP composite, Super P, and PVDF were weighed in a mortar according to the mass ratio of 7:2:1, and then ground and mixed. The milled electrode slurry was evenly coated on the collector (clean aluminum foil) and placed in a vacuum drying oven. The drying conditions were 110°C/12 h. The dried electrodes were rolled with a roller press. The area of each working electrode is 1.15 cm^2^ and the loading of active material is about 2 mg/cm^2^.

#### 2.3.2 Preparation of LiMn_2_O_4_ (LMO) cathode material

LiMn_2_O_4_, Super P, and PVDF were mixed in the mass ratio of 75:15:10 and ground well. After that, an appropriate amount of NMP was added to it, and the grinding was continued for a certain time, and the ground slurry was evenly coated on the stainless steel. The coated stainless steel with electrode material was dried in a vacuum drying oven at 100°C for 12 h. The dried electrodes were rolled and then sliced. The area of the cut single electrode is 1.15 cm^2^ and the loading of its active material is about 2 mg/cm^2^.

#### 2.3.3 Assembly of lithium-ion battery

Organic lithium-ion battery assembly: To assemble the organic lithium-ion battery, the button cell (CR2016 type) was assembled in an Ar-filled glove box with both H_2_O/O_2_ concentrations less than 0.1 ppm, using lithium cells as counter electrodes, the diaphragm was a common celgard 2,400 diaphragm, and the electrolyte used was 1 M LiPF6 organic solution, in which the volume ratio of solvent EC:DEC:DMC was 1:1: 1.

## 3 Results and discussion

### 3.1 Material characterization

#### 3.1.1 XRD analysis

**FIGURE 1:**
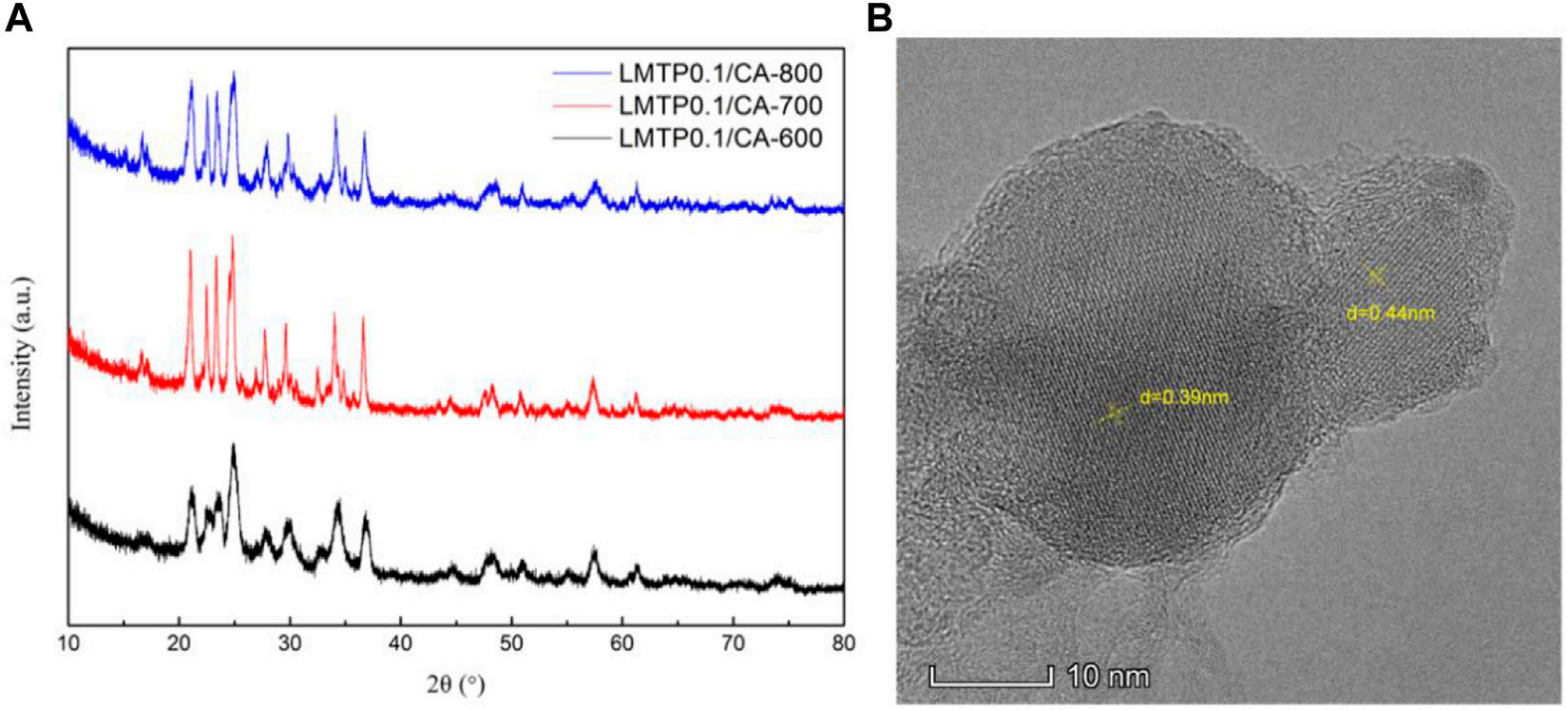
XRD plots of LMTP0.1/CA composites made at 600°C, 700°C and 800°C **(A)**; TEM image of LMTP0.1/CA-700 material **(B)**.

**FIGURE 2:**
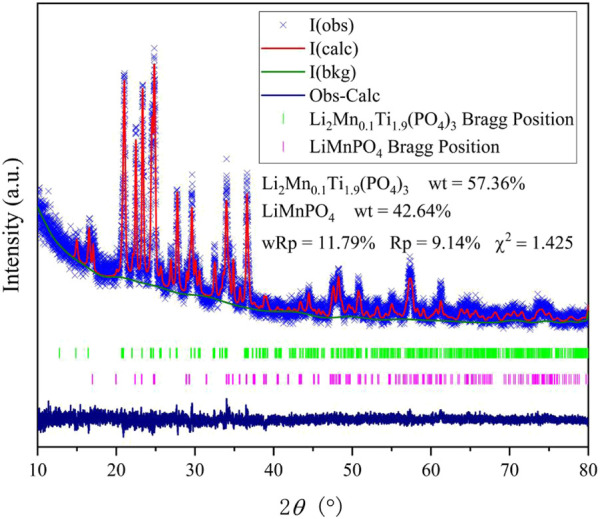
Rietveld plot for Li_2_Mn_0.1_Ti_1.9_(PO_4_)_3_ and LiMnPO_4_ (observed, calculated, back grand, difference, Bragg position of XRD peaks).

**FIGURE 3:**
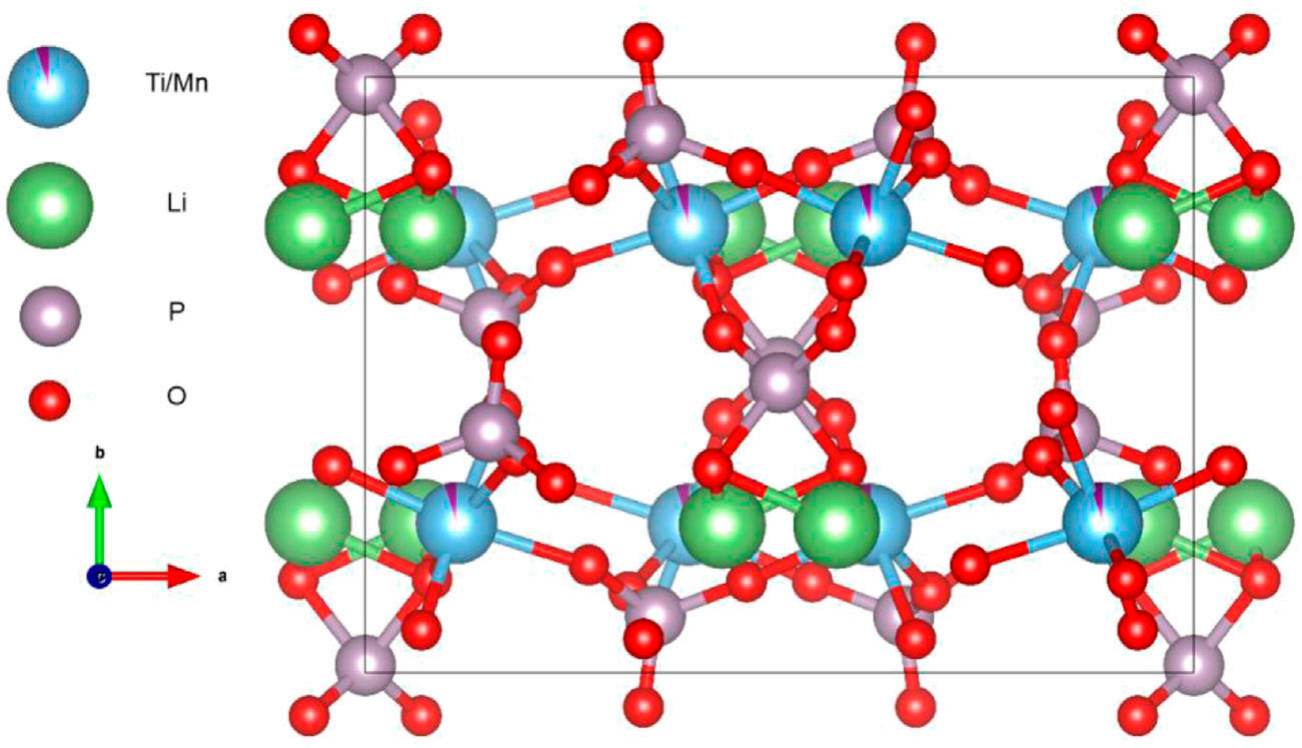
Crystal structure of Li_2_Mn_0.1_Ti_1.9_(PO_4_)_3_, Ti atom and Mn atom occupied 90% and 10% ratio of the same position, respectively.

The XRD plots of the materials obtained by sintering at different temperatures are shown in Figure 1A. From Figure 1A, it can be seen that the temperature has a strong influence on the crystal structure of the material, and the crystallinity of the material increases when the temperature increases, but the crystallinity of the material does not continue to increase when the temperature is increased to a certain level, and the material obtained by sintering at 700°C has the best crystallinity, with the positions of the main diffraction peaks at 21.1°, 22.6°, 23.4°, and 24.9°. In order to analysis the structure of LMTP, the XRD data of LMTP-700 was selected as the source of the refinement data. The refined data show that LMTP-700 is a complex composed of Mn-doped Li_2_Mn_0.1_Ti_1.9_(PO_4_)_3_ and LiMnPO_4_, and their mass fractions are 57.36% and 42.64%, respectively. Among them, the space group of Li_2_Mn_0.1_Ti_1.9_(PO_4_)_3_ is Pbcn and the corresponding lattice parameter *a* = 11.9372 Å, *b* = 8.5409 Å, *c* = 8.5979 Å, *α* = *β* = *γ* = 90°, *V* = 876.59 Å^3^ and *Z* = 4). Rietveld refinement was performed (Toby, 2001), and the confidence factors are R_wp_ = 11.79%, R_p_ = 9.14%, and χ^2^ = 1.425. Rietveld plot for Li_2_Mn_0.1_Ti_1.9_(PO_4_)_3_ and LiMnPO_4_ (observed, calculated, background, and difference PXRD profiles along with Bragg peak positions) were shown in Figure 2. The refinement results show that the Mn atom doping in it occupies 10% of the Ti atom positions (Momma & Izumi, 2011) in Figure 3. Table 1 shows the refined lattice parameters.

**TABLE 1:** Refined lattice parameters along with R-factors.

Lattice parameters	Crystal data using GSAS
Crystal system	orthorhombic
Space group	Pbcn
*a*	11.9371 (8)Å
*b*	8.5409 (6)Å
*c*	8.5979 (6)Å
*α*	90.0°
*β*	90.0°
*γ*	90.0°
Cell volume	876.59 (11)Å^3^
Z	4
R_wp_	11.79%
R_p_	9.14%
χ^2^	1.425

#### 3.1.2 SEM analysis

**FIGURE 4:**
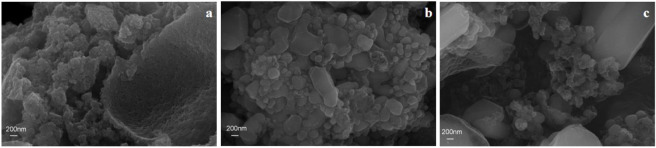
SEM images of LMTP0.1/CA composites sintered at 600°C **(A)**, 700°C **(B)** and 800°C **(C)**.

SEM characterization was performed for the LMTP0.1/CA materials made at different temperatures to observe their morphology. Figure 4A shows the SEM images at 600°C, 700°C, and 800°C at the same size (200 nm), respectively. From Figure 4A, it can be seen that the LMTP0.1/CA-600 material shows a spherical shape, but there are still some incomplete sintered particles and the crystallinity of the material is low. From Figure 4B, it can be seen that the LMTP0.1/CA-700 material mostly presents spherical particles and is more uniform, but there are still some larger particles. From Figure 4C, it can be seen that the LMTP0.1/CA-800 material shows variable particle shapes and uneven sizes. Therefore, it can be seen that the shape of the material is relatively more regular with small spherical shape at the sintering temperature of 700°C.

#### 3.1.3 TEM analysis

To further characterize the specific morphology of LMTP0.1/CA-700 material, TEM was used to characterize the structure of the material, as shown in Figure 1B As can be seen from Figure 1B, the material has obvious lattice stripes, indicating a high crystallinity of the material. Using the analysis software, the lattice stripe spacing was measured as 0.39 nm and 0.44 nm, respectively. 2θ, calculated according to the Bragg formula, was obtained as 22.77° and 20.16°, which was in agreement with the XRD results.

#### 3.1.4 XPS analysis

**FIGURE 5:**
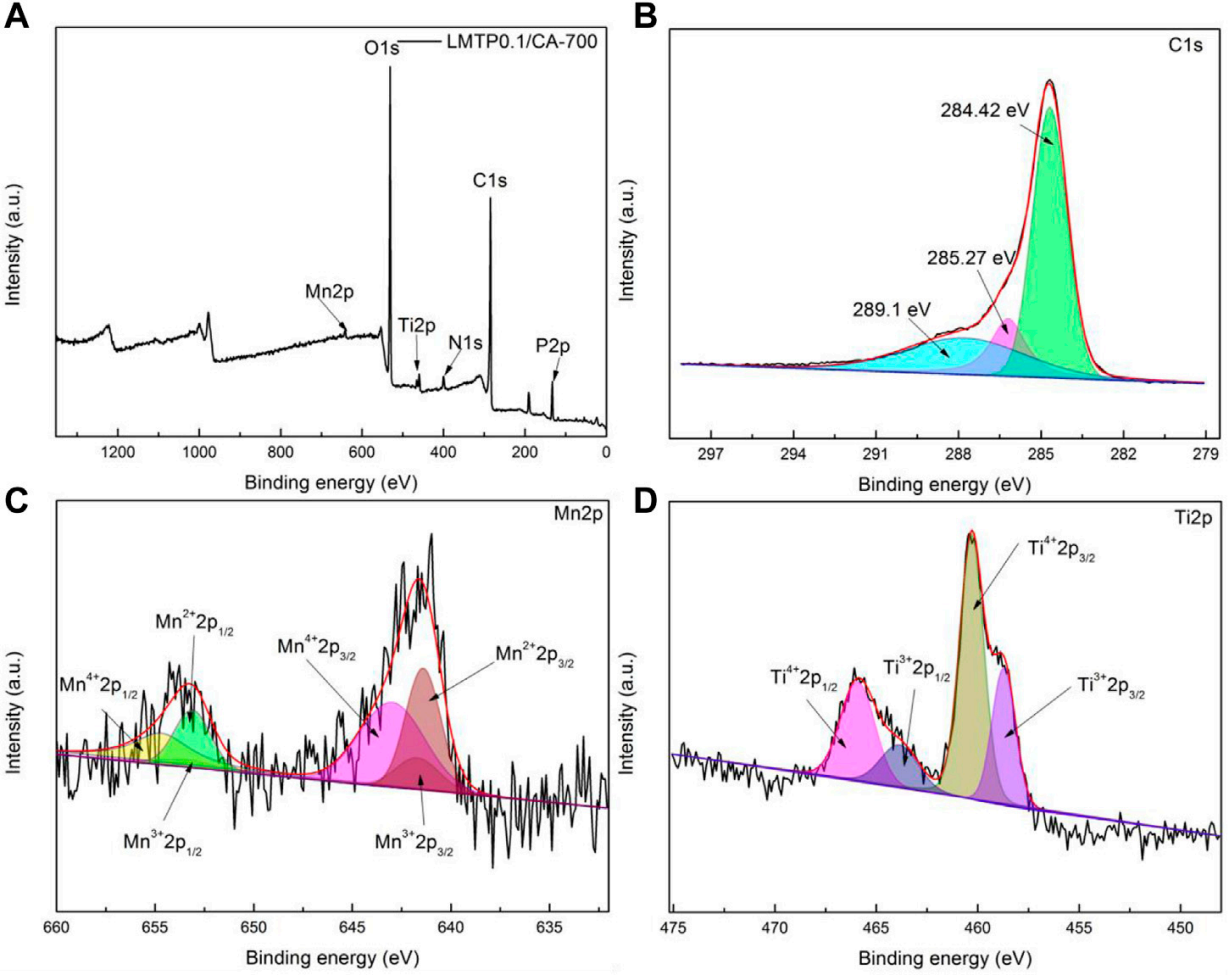
XPS diagram of LMTP0.1/CA-700 material **(A)**; C_1s_ **(B)**; Mn_2p_ **(C)**; Ti_2p_ **(D)**.

To determine the valence state of each element in the material, XPS analysis of the fabricated LMTP0.1/CA-700 material was performed, as shown in Figure 5A. The surface electronic state and chemical composition of the material can be obtained according to the analysis of the experimental results, and the signals of C, Ti, Mn, and P elements are detected, but the signal peak of Mn is weaker, which may be due to the low content of Mn elements in the material compared with the content of other elements. Figure 5B shows that the material exhibits the strong peaks of C_1s_, with a total of three strong peaks (289.1 eV, 285.27 eV, 284.42 eV)corresponding to the three chemical bonds C=O (288.40 eV), C-O (286.14 eV) and C-C or C=C (284.80 eV) (Shchukarev & Korolkov, 2004; Liu et al., 2017; Wang L. et al, 2020), and the content of C-C is higher in terms of peak area. Figure 5C shows the signal peaks of Mn_2p_, which has two sets of peaks consisting of three sub-peaks for each set of peaks found by fitting the sub-peaks. In the higher binding energy region Mn2p_1/2_ shows peaks for Mn^4+^, Mn^3+^ and Mn^2+^ at 654.88 eV, 653.38 eV and 653.18 eV positions, respectively. Similarly, in the lower binding energy region Mn2p_3/2_ shows peaks for Mn^4+^, Mn^3+^ and Mn^2+^ at positions 642.98 eV, 642.78 eV and 641.48 eV, respectively. It exhibits that Mn^2+^ and Mn^4+^ are predominant. This is in general agreement with what is described in the literature (Biesinger et al., 2011). Two distinct peaks of Ti_2p_ can be seen in Figure 5D The fitting results of the XPS peaks of Ti elements showed four sub-peaks at the binding energy positions Ti^4+^2p_1/2_ (465.88 eV), Ti^3+^2p_1/2_ (463.88 eV), Ti^4+^2p_3/2_ (460.38 eV) and Ti^3+^2p_3/2_ (456.68 eV) (Ding et al., 2021; Torres-Ceron et al., 2021), respectively. The integration of the peak area reveals that Ti^4+^ is present in higher concentrations.

#### 3.1.5 BET analysis

**FIGURE 6:**
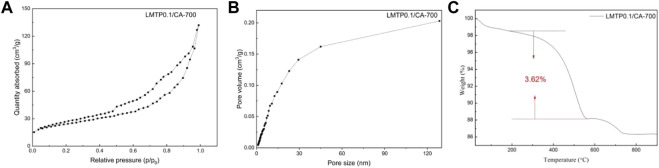
**(A)** N_2_ adsorption isotherm curves and **(B)** pore size distribution curves of LMTP0.1/CA-700; **(C)** Thermogravimetric plots of LMTP0.1/CA-700 composites.

Figures 6A,B shows the adsorption isotherms and pore size distributions of LMTP0.1/CA materials with anhydrous citric acid as carbon source and synthesis temperatures of 700°C. It can be seen from the plots that the pore sizes of the materials are mainly distributed around 0–25 nm, indicating that the synthesized materials are mesoporous in structure. The measured specific surface area of LMTP/CA-700 is 96.07 m^2^/g (Figure 6A), corresponding to a porosity of 0.20 cm^2^/g (Figure 6B). In comparison, the LMTP0.1/CA-700 material has a larger specific surface area and is more suitable for Li^+^ de-embedding.

#### 3.1.6 TGA analysis

To detect the carbon content in the material, TGA was performed on the material, and the obtained data are shown in Figure 6C. Figure 6C shows the thermogram of LMTP0.1/CA-700, and the reduction is 3.62% of carbon content from 300°C to 900°C according to the thermogram result analysis. From 28°C to 200°C, the reduction could be the water content in the material. According to the experimental results, too much carbon coating is not beneficial to improve the electrochemical performance of the material, which may be due to the fact that too much carbon content leads to a decrease in the mass of the active material, which in turn leads to poor electrochemical performance of the material.

### 3.2 Electrochemical performance test

To investigate the effect of sintering temperature on the electrochemical performance of LMTP0.1/CA materials, cells were assembled separately to test their electrochemical performance.

**FIGURE 7:**
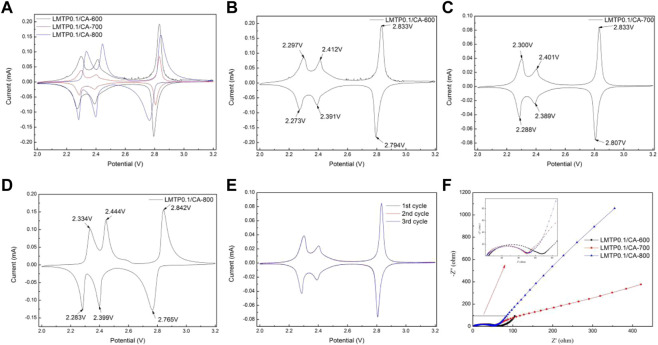
**(A-D)** CV plots of LMTP0.1/CA composites sintered at different temperatures; **(E)** CV plots of the first three cycles of the LMTP0.1/CA-700 composite; **(F)** AC impedance test plots of LMTP0.1/CA material sintered at different temperatures.

The electrochemical reversibility of the LMTP0.1/CA electrode materials sintered at different temperatures was tested separately using cyclic voltammetry, as shown in Figure 7. The test voltage range of the composites was from 2.0 to 3.2 V (vs Li^+^/Li) and the scan rate was 0.1 mV/s. As shown in Figure 7A, three pairs of redox peaks were present in the LMTP0.1/CA composites at the specified voltage range (2.0–3.2 V). Figures 7B–D shows the specific locations of the redox peaks for the LMTP0.1/CA composite sintered at different temperatures using anhydrous citric acid as the carbon source, respectively. The overpotentials of the three materials, LMTP0.1/CA-600, LMTP0.1/CA-700, and LMTP0.1/CA-800, can be calculated as shown in Table 2. In comparison to sintered materials sintered at 600°C (24 mV, 21 mV, 39 mV) and 800°C (51 mV, 45 mV, 77 mV), 700°C sintered composites (12 mV, 12 mV, 26 mV) exhibit a reduced overpotential per pair of redox peaks with a greater reversibility in electrochemistry.

**TABLE 2:** Calculation of overpotential for LMTP0.1/CA composites sintered at different temperatures.

	LMTP0.1/CA-600			LMTP0.1/CA-700			LMTP0.1/CA-800		
V_1_/V	2.297	2.412	2.833	2.300	2.401	2.833	2.334	2.444	2.842
V_2_/V	2.273	2.391	2.794	2.288	2.389	2.807	2.283	2.399	2.765
ΔV/V	0.024	0.021	0.039	0.012	0.012	0.026	0.051	0.045	0.077

To further understand the cycle reversibility performance of the LMTP0.1/CA-700 composite, the material was tested for 3 cycles under the same conditions, and the results are shown in Figure 7E, where the scan rate was set to 0.1 mV/s and the voltage range was set to 2.0 V–3.2 V (vs Li^+^/Li). From Figure 7E, it can be seen that there are three pairs of redox peaks for the LMTP0.1/CA-700 composite, and the main redox peak is at 2.83/2.80 V. The results show that the reactions occurring in this voltage interval of the material are all reversible. The redox peaks of each cycle nearly coincide in three cycles, which further indicates that the LMTP0.1/CA-700 material has good cycle reversibility and high cycle stability.

Figure 7F shows the AC impedance plots of cells with LMTP0.1/CA material sintered at different temperatures as the positive electrode and lithium sheet as the counter electrode. The impedance diagram mainly consists of two parts, which are a half circle in the mid-high frequency region and a straight line in the low frequency region. As can be seen from Figure 7F, the half-circle area in the impedance diagram of LMTP0.1/CA-600 material is much larger than that of LMTP0.1/CA-700 and LMTP0.1/CA-800, while in the low-frequency straight line area but the slope of the straight line of LMTP0.1/CA-800 material is larger than that of LMTP0.1/CA-700, according to the idea that the smaller the slope of the straight line, the better the ion diffusion of the material. Based on the idea that the smaller the linear slope, the better the ion diffusion performance of the material, it can be shown that the charge transfer resistance of LMTP0.1/CA-700 is smaller, while the Li^+^ diffusion resistance is reduced. As expected, the increase in temperature is beneficial to the improvement of the material properties, however, too high temperature may damage the structure of the material, which is not conducive to the enhancement of the electrochemical properties of the material.

**FIGURE 8:**
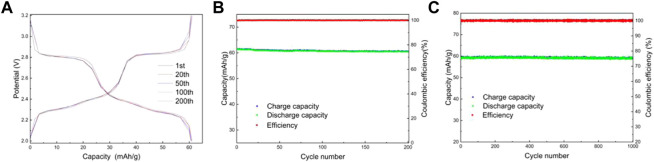
Charge and discharge curves of LMTP0.1/CA-600 **(A)**, Electrochemical performance of LMTP0.1/CA-600 **(B)** cycled for 200 cycles at a current density of 200 mA/g; Capacity/efficiency-cycles plot of LMTP0.1/CA-600cycled for 1,000 turns at a current density of 1,000 mA/g **(C)**.

Lithium-ion batteries were assembled using lithium sheets as counter electrodes. Figures 8A,B shows the charge/discharge curves and capacity/efficiency-voltage plots of LMTP0.1/CA-600 for 200 cycles of constant cycling (at a current density of 200 mA/g). As can be seen from the figure, the specific capacity of LMTP0.1/CA-600 at the 1st, 20th, 50th, 100th, and 200th cycles of discharge is 61.5 mAh/g, 61.1 mAh/g, 60.8 mAh/g, 60.6 mAh/g, and 60.5 mAh/g, respectively, and the capacity retention rate of the material is 98%. It indicates that the material has high cycling stability. Figure 8C shows the electrochemical performance of LMTP0.1/CA-600 for 1,000 cycles at a constant cycle (at a current density of 1,000 mA/g). As can be seen from the graph, the first cycle discharge specific capacity of the cell is 59.1 mAh/g. As the 1,000 charge/discharge cycles proceed, the material remains at about 58.8 mAh/g and the Coulomb efficiency remains at about 99.8%. The capacity of the LMTP0.1/CA-600 material decays by 0.6% at high current densities, so the material maintains good cycling stability.

**FIGURE 9:**
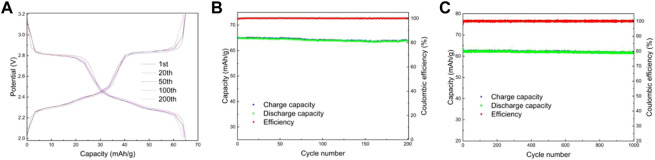
Charge and discharge curves of LMTP0.1/CA-700 **(A)**, Electrochemical performance of LMTP0.1/CA-700 **(B)** cycled for 200 cycles at a current density of 200 mA/g; Capacity/efficiency-cycles plot of LMTP0.1/CA-700cycled for 1,000 turns at a current density of 1,000 mA/g **(C)**.

Figures 9A,B shows the electrochemical performance of LMTP0.1/CA-700 material for 200 cycles at a current density of 200 mA/g. From Figure 9A, it can be seen that the material has a smooth charging and discharging plateau with a long plateau time. Figure 9A shows the capacity/efficiency-cycles plot of LMTP0.1/CA-700 material. The specific discharge capacities of LMTP0.1/CA-700 at the 1st, 20th, 50th, 100th, and 200th cycles are 65.2 mAh/g, 64.8 mAh/g, 64.8 mAh/g, 64.1 mAh/g, and 63.8 mAh/g, respectively. , the capacity retention rate of the material was 97%. This indicates that the material has high cycling stability. Figure 9B shows the electrochemical performance of LMTP0.1/CA-700 for 1,000 cycles at constant cycle (current density of 1,000 mA/g). As can be seen from the graph, the initial discharge specific capacity of the battery is 63.7 mAh/g, and the material remains at about 61.4 mAh/g after 1,000 cycles. As the charge and discharge proceed, the material keeps a high specific capacity and the Coulomb efficiency has been maintained above 99%. It indicates that LMTP0.1/CA-700 still maintains high specific capacity of discharge and excellent cycling performance under high current density. Comparing with Figure 9C, it also indicates that the elevated temperature promotes the increase of material crystallinity, which improves the electrochemical properties of the material.

**FIGURE 10:**
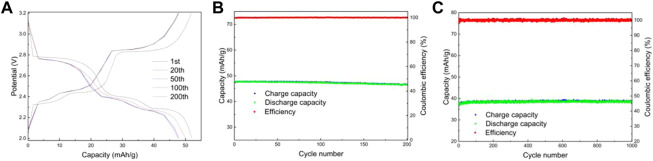
Charge and discharge curves of LMTP0.1/CA-800 **(A)**, Electrochemical performance of LMTP0.1/CA-800 **(B)** cycled for 200 cycles at a current density of 200 mA/g; Capacity/efficiency-cycles plot of LMTP0.1/CA-800cycled for 1,000 turns at a current density of 1,000 mA/g **(C)**.

Figures 10A,B shows the electrochemical performance of LMTP0.1/CA-800 material cycled for 200 turns at a current density of 200 mA/g. From Figure 10A, it can be seen that there are obvious three sets of plateaus in the material, which is consistent with the CV results, but the plateau time is shorter. Figure 10B shows the capacity/efficiency-cycles plot of LMTP0.1/CA-700 material. The discharge specific capacities of LMTP0.1/CA-700 at the 1st, 20th, 50th, 100th, and 200th cycles are 47.8 mAh/g, 47.7 mAh/g, 47.6 mAh/g, 47.4 mAh/g, and 46.5 mAh/g, respectively, the capacity retention of the material was 97%. It indicates that the cycling stability of the material is good, but the specific capacity of the material is poor. Figure 10C shows the electrochemical performance of LMTP0.1/CA-800 for 1,000 cycles at a constant cycle (1,000 mA/g current density). As can be seen from the graph, the battery starts with a discharge specific capacity of 39.8 mAh/g, and after 1,000 cycles, the material can be maintained at about 38.6 mAh/g. As the charge and discharge proceed, the capacity of the material decays by about 3%, while the Coulomb efficiency has been maintained at about 99%.

From the above data, it can be seen that the LMTP0.1/CA material can maintain good cycle reversibility and high Coulomb efficiency in both charge and discharge constant cycle tests at high current density. However, the electrochemical performance of the LMTP/CA-700 material is better compared to the remaining two materials at temperature due to its highest discharge specific capacity. The material produced at 600°C and 800°C has a lower specific discharge capacity when the current density becomes higher. Therefore, 700°C is the best preparation condition for this material.

**FIGURE 11:**
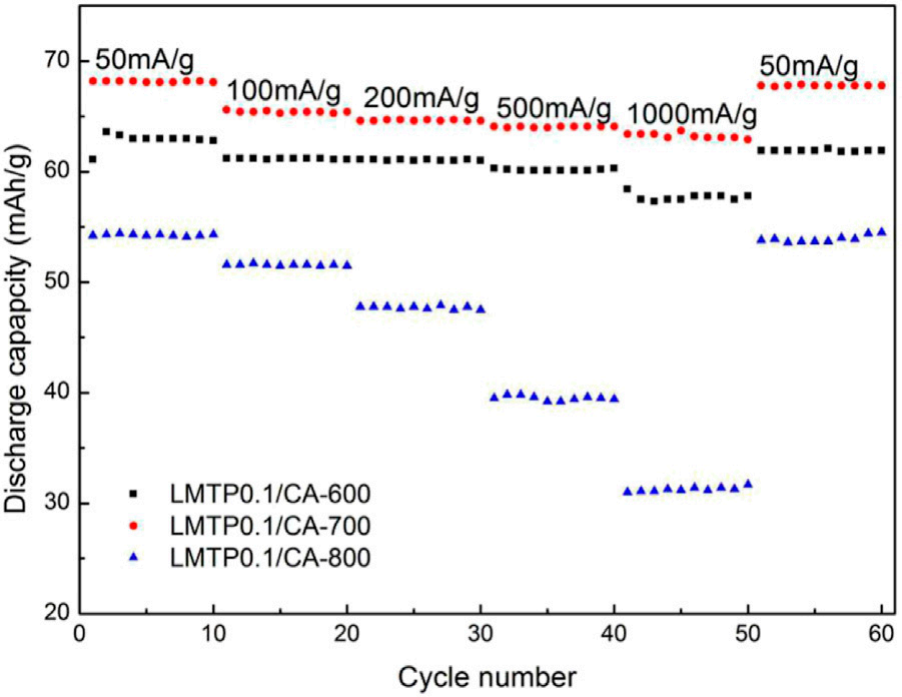
Multiplicative properties of LMTP0.1/CA materials sintered at different temperatures.

Figure 11 shows the cycling plots of LMTP0.1/CA materials sintered at different temperatures at different current densities (50 mA/g, 100 mA/g, 200 mA/g, 500 mA/g, 1,000 mA/g). As can be seen from the graphs, the charge/discharge specific capacity of LMTP0.1/CA-700 remains the highest and decays less when the current density is changed, indicating that the material also has the best cycling stability. In comparison, the charge/discharge capacity of LMTP0.1/CA-600 is slightly reduced. The charge/discharge specific capacity of LMTP0.1/CA-800 is the lowest, and the material attenuation is extremely obvious when the current density is changed. When the current density reaches 1,000 mA/g, the charge/discharge specific capacity is only 32.2 mAh/g, indicating that the material has extremely poor cycle reversibility. This agrees with the results obtained from previous electrochemical performance tests, indicating that the material sintered at 700°C has the best electrochemical performance.

Looking at the electrochemical performance of all batteries with synthetic materials as the positive electrode and lithium flakes as the negative electrode, it can be found that the LMTP0.1/CA material sintered at 700°C with anhydrous citric acid as the carbon source has the best electrochemical performance and can maintain a more stable capacity even at higher current densities. This specific capacity still has a lot of optimizing space compared to that of the commercial battery material LiFePO_4_, however, the scientific contribution of this Mn-doped phosphate series materials provides a direction for more in-depth and extensive research in order to make a breakthrough in the mechanism and find the most optimized technological parameters required for better electrochemical performance.

## 4 Conclusion

In this paper, LMTP0.1/CA composites were prepared by spray drying method and subjected to conventional characterization and electrochemical performance tests. The XRD and SEM results showed that the LMTP0.1/CA-700 material has good crystallinity, and the SEM patterns showed obvious spherical particles. The BET showed that the pore size of the material is of micro-nanometer level, which facilitates the entry and exit of Li^+^. The half-cells were assembled with the synthesized materials as the positive electrode, and the CV curves showed three reaction platforms for each of the synthesized materials. Testing the cells with the same LAND test procedure (200 mA/g for 200 cycles), the LMTP0.1/CA-700 material has a discharge specific capacity of about 65 mAh/g. The capacity decayed by only 3% during the cycle.

The full cells with 600°C and 700°C sintered materials as negative electrodes decayed less under the same test conditions (200 mA/g for 200 cycles), but the specific capacity of LMTP0.1/CA-700 (60 mAh/g) was about 20 mAh/g higher than that of LMTP0.1/CA-600 (40 mAh/g), while LMTP0.1/CA- 800 has a higher initial specific capacity, but decays faster during cycling, decaying by 12%. It indicates that LMTP0.1/CA-700 material has better performance when used as anode material for water-based batteries. Through the above structural characterization and electrochemical tests, the material made by sintering at 700°C has the best morphology and the best electrochemical performance. This may be due to the poor crystallinity of the material when the sintering temperature is too low and the internal collapse of the material structure when the sintering temperature is too high. Future research will focus on the doping element type, ratio and preparation control condition, which also provide a potential research idea for the better battery material of phosphate system, and also show from the side that the doping element will have a significant contribution to the electrochemical performance of the battery material, and this series material will also have an extensive electrochemical application value.

## Data Availability

The raw data supporting the conclusion of this article will be made available by the authors, without undue reservation.

## References

[B1] Biesinger M. C. Payne B. P. Grosvenor A. P. Lau L. W. M. Gerson A. R. Smart R. S. C. (2011). Resolving surface chemical states in XPS analysis of first row transition metals, oxides and hydroxides: Cr, Mn, Fe, Co and Ni. Appl. Surf. Sci. 257 (7), 2717–2730. 10.1016/j.apsusc.2010.10.051

[B2] Capsoni D. Bini M. Ferrari S. Mustarelli P. Massarotti V. Mozzati M. C. (2010). Structural, spectroscopic, and electrical features of undoped and Mn-doped LiTi_2_(PO_4_)_3_ . J. Phys. Chem. C 114 (32), 13872–13878. 10.1021/jp104571a

[B3] Catti M. (2001). A mixed α/β superstructure in NASICON ionic conductors: Neutron diffraction study of Li_2_FeTi(PO_4_)_3_ and Li_2_FeZr(PO_4_)_3_ . J. Solid State Chem. 156 (2), 305–312. 10.1006/jssc.2000.8999

[B4] Chen H. Yang X. Lv P. Tian P. Wan S. Liu Q. (2022). Mn-doped FeS with larger lattice spacing as advance anode for sodium ion half/full battery. Chem. Eng. J. 450, 137960. 10.1016/j.cej.2022.137960

[B5] Ding J. M. Zhu P. F. Li Z. Q. Wang Z. Y. Ai L. Zhao J. F. (2021). Synthesis, electronic structure and upconversion photoluminescence of langbeinite-type K_2_TiYb (PO_4_)_3_ microcrystals. OPTIK 244, 167549. 10.1016/j.ijleo.2021.167549

[B6] Gao X. P. Yang H. X. (2010). Multi-electron reaction materials for high energy density batteries. Energy Environ. Sci. 3 (2), 174–189. 10.1039/b916098a

[B7] Giarola M. Sanson A. Tietz F. Pristat S. Dashjav E. Rettenwander D. (2017). Structure and vibrational dynamics of NASICON-type LiTi_2_(PO_4_)_3_ . J. Phys. Chem. C 121 (7), 3697–3706. 10.1021/acs.jpcc.6b11067

[B8] Guo Z. Qin X. Xie Y. Lei C. Wei T. Zhang Y. (2022). Advanced NASICON-type LiTi2(PO4)3 as electrode materials for lithium-ion batteries. Chem. Phys. Lett. 806, 140010. 10.1016/j.cplett.2022.140010

[B9] Gupta R. Saha S. Tomar M. Sachdev V. K. Gupta V. (2017). Effect of manganese doping on conduction in olivine LiFePO_4_ . J. Mater. Science-Materials Electron. 28 (7), 5192–5199. 10.1007/s10854-016-6175-9

[B10] He S. Xu Y. (2019). Hydrothermal-assisted solid-state reaction synthesis of high ionic conductivity Li_1+x_Al_x_Ti_2−x_(PO_4_)_3_ ceramic solid electrolytes: The effect of Al^3+^ doping content. Solid State Ionics 343, 115078. 10.1016/j.ssi.2019.115078

[B11] He Z. Jiang Y. Meng W. Zhu J. Liu Y. Dai L. (2016). Advanced LiTi_2_(PO_4_)_3_@N-doped carbon anode for aqueous lithium ion batteries. Electrochimica Acta 222, 1491–1500. 10.1016/j.electacta.2016.11.128

[B12] He Z. Jiang Y. Zhu J. Wang H. Li Y. Zhou H. (2018). N-doped carbon coated LiTi_2_(PO_4_)_3_ as superior anode using PANi as carbon and nitrogen bi-sources for aqueous lithium ion battery. Electrochimica Acta 279, 279–288. 10.1016/j.electacta.2018.05.096

[B13] Jiang Z. Li Y. Han C. He Z. Gong Y. Meng W. (2019a). Endowing LiTi_2_(PO_4_)_3_/C with excellent electrochemical performances through rational crystal doping. Ceram. Int. 45 (17), 23406–23410. 10.1016/j.ceramint.2019.08.043

[B14] Jiang Z. Li Y. Han C. He Z. Meng W. Dai L. (2019b). K doping on Li site enables LiTi_2_(PO_4_)_3_/C excellent lithium storage performance. Solid State Ionics 341, 115036. 10.1016/j.ssi.2019.115036

[B15] Jin Y. Zhang T. Zhang M. (2022). Advances in intelligent regeneration of cathode materials for sustainable lithium‐ion batteries. Adv. Energy Mater. 12 (36), 2201526. 10.1002/aenm.202201526

[B16] Kee Y. Lee S. S. Yun H. (2011) The mixed-valent titanium phosphate, Li_2_Ti_2_(PO_4_)_3_, dilithium dititanium(III/IV) tris(orthophosphate). Acta Crystallogr. Sect. E 67 (9), i49. 10.1107/S1600536811031606 PMC3200769 22065701

[B17] Kwatek K. Świniarski M. Nowiński J. L. (2018). The Li^+^ conducting composite based on LiTi_2_(PO_4_)_3_ and Li_3_BO_3_ glass. J. Solid State Chem. 265, 381–386. 10.1016/j.jssc.2018.06.028

[B18] Liang Y. Peng C. Kamiike Y. Kuroda K. Okido M. (2019). Gallium doped NASICON type LiTi_2_(PO_4_)_3_ thin-film grown on graphite anode as solid electrolyte for all solid state lithium batteries. J. Alloys Compd. 775, 1147–1155. 10.1016/j.jallcom.2018.10.226

[B19] Lin Z. P. Zhao Y. M. Zhao Y. J. (2011). First-principles studies of Mn-doped LiCoPO_4_ . Chin. Phys. B 20 (1), 018201. 10.1088/1674-1056/20/1/018201

[B20] Liu S. Xin Z. J. Lei Y. J. Yang Y. Yan X. Y. Lu Y. B. (2017). Thin copper-based film for efficient electrochemical hydrogen production from neutral aqueous solutions. ACS Sustain. Chem. Eng. 5 (9), 7496–7501. 10.1021/acssuschemeng.7b01646

[B21] Luo H. Tang Y. Xiang Z. Wu P. Li Z. (2020). Cl(-) doping strategy to boost the lithium storage performance of lithium titanium phosphate. Front. Chem. 8, 349. 10.3389/fchem.2020.00349 32528923 PMC7247843

[B22] Luo Y. Jiang X. Yu Y. Liu L. Lin X. Wang Z. (2023). Enhancement of electrical properties of LiTi_2_(PO_4_)_3_ ceramics via trivalent cation doping and microstructure regulation strategies. Solid State Ionics 390, 116111. 10.1016/j.ssi.2022.116111

[B23] Momma K. Izumi F. (2011). VESTA 3for three-dimensional visualization of crystal, volumetric and morphology data. J. Appl. Crystallogr. 44 (6), 1272–1276. 10.1107/s0021889811038970

[B24] Pang J. Kuang Q. Zhao Y. Han W. Fan Q. (2018). A comparative study of LiTi_2_(P_8/9_V_1/9_O_4_)_3_ and LiTi_2_(PO_4_)_3_: Synthesis, structure and electrochemical properties. Electrochimica Acta 260, 384–390. 10.1016/j.electacta.2017.12.073

[B25] Patoux S. Rousse G. Leriche J. B. Masquelier C. (2004). Crystal structure and lithium insertion properties of orthorhombic Li_2_TiFe(PO_4_)_3_ and Li_2_TiCr(PO_4_)_3_ . Solid State Sci. 6 (10), 1113–1120. 10.1016/j.solidstatesciences.2004.07.028

[B26] Shchukarev A. V. Korolkov D. V. (2004). XPS study of group IA carbonates. CENTRAL Eur. J. Chem. 2 (2), 347–362. 10.2478/BF02475578

[B27] Shi Z. Xu W. Ru Q. Zheng M. Zhang J. Chi-Chun Ling F. (2021). Mn-doped K_0.23_V_2_O_5_ nanobelts as cathode materials for high performance flexible all-in-one zinc ion batteries. J. Power Sources 516, 230699. 10.1016/j.jpowsour.2021.230699

[B28] Song X. Yu W. Zhou S. Zhao L. Li A. Wu A. (2023). Enhancement of Mn-doped LiPON electrolyte for higher performance of all-solid-state thin film lithium battery. Mater. Today Phys. 33, 101037. 10.1016/j.mtphys.2023.101037

[B29] Sradhasagar S. Mallick S. Rath A. Pati S. Roy A. (2023). Role of Fe^3+^ doping vis-à-vis secondary phases on the electrical transport of LiTi_2_(PO_4_)_3_ solid electrolyte. Mater. Today Commun. 35, 105621. 10.1016/j.mtcomm.2023.105621

[B30] Sun D. Xue X. Tang Y. Jing Y. Huang B. Ren Y. (2015). High-rate LiTi_2_(PO_4_)_3_@N-C composite via Bi-nitrogen sources doping. ACS Appl. Mater Interfaces 7 (51), 28337–28345. 10.1021/acsami.5b08697 26633580

[B31] Sun L. Yao Y. Dai L. Jiao M. Ding B. Yu Q. (2022). Sustainable and high-performance Zn dual-ion batteries with a hydrogel-based water-in-salt electrolyte. Energy Storage Mater. 47, 187–194. 10.1016/j.ensm.2022.02.012

[B32] Toby B. H. (2001). EXPGUI, a graphical user interface forGSAS. J. Appl. Crystallogr. 34 (2), 210–213. 10.1107/s0021889801002242

[B33] Torres-Ceron D. A. Amaya-Roncancio S. Riva J. S. Vargas-Eudor A. Escobar-Rincon D. Restrepo-Parra E. (2021). Incorporation of P^5+^ and P^3-^ from phosphate precursor in TiO_2_:P coatings produced by PEO: XPS and DFT study. Surf. COATINGS Technol. 421, 127437. 10.1016/j.surfcoat.2021.127437

[B34] Wang H. Zhang H. Cheng Y. Feng K. Li X. Zhang H. (2017). Rational design and synthesis of LiTi_2_(PO_4_)_3−x_F_x_ anode materials for high-performance aqueous lithium ion batteries. J. Mater. Chem. A 5 (2), 593–599. 10.1039/c6ta08257b

[B35] Wang L. Sofer Z. Pumera M. (2020a). Will any crap we put into graphene increase its electrocatalytic effect? ACS Nano 14 (1), 21–25. 10.1021/acsnano.9b00184 31934742

[B36] Wang Y. Q. Sun X. R. Xiao R. J. Chen L. Q. (2020b). Computational screening of doping schemes for LiTi_2_(PO_4_)_3_ as cathode coating materials. Chin. Phys. B 29 (3), 038202. 10.1088/1674-1056/ab7186

[B37] Weng G. M. Simon Tam L. Y. Lu Y. C. (2017). High-performance LiTi_2_(PO_4_)_3_ anodes for high-areal-capacity flexible aqueous lithium-ion batteries. J. Mater. Chem. A 5 (23), 11764–11771. 10.1039/c7ta00482f

[B38] Yin S. C. Grondey H. Strobel P. Anne M. Nazar L. F. (2003). Electrochemical property: Structure relationships in monoclinic Li_3-y_V_2_(PO_4_)_3_ . J. Am. Chem. Soc. 125 (34), 10402–10411. 10.1021/ja034565h 12926965

[B39] Zeng H. Liu D. Zhang Y. See K. A. Jun Y. S. Wu G. (2015). Nanostructured Mn-doped V_2_O_5_ cathode material fabricated from layered vanadium jarosite. Chem. Mater. 27 (21), 7331–7336. 10.1021/acs.chemmater.5b02840

[B40] Zhang P. Matsui M. Takeda Y. Yamamoto O. Imanishi N. (2014). Water-stable lithium ion conducting solid electrolyte of iron and aluminum doped NASICON-type LiTi_2_(PO_4_)_3_ . Solid State Ionics 263, 27–32. 10.1016/j.ssi.2014.04.017

[B41] Zhang X. Liu Q. Liu S. Wang E. (2023). Manganese-doped cobalt spinel oxide as bifunctional oxygen electrocatalyst toward high-stable rechargeable Zn-air battery. Electrochimica Acta 437, 141477. 10.1016/j.electacta.2022.141477

[B42] Zhao W. Chen L. Xue R. Min J. Cui W. (1994). Ionic conductivity and luminescence of Eu^3+^-doped LiTi_2_(PO_4_)_3_ . Solid State Ionics 70-71, 144–146. 10.1016/0167-2738(94)90299-2

[B43] Zhou Z. Xiang A. Xia M. Zhou N. (2018). Advanced LiTi_2_(PO_4_)_3_ anode with high performance for aqueous rechargeable lithium battery. Ceram. Int. 44 (17), 21599–21606. 10.1016/j.ceramint.2018.08.240

